# *LaminaRGeneVis*: A Tool to Visualize Gene Expression Across the Laminar Architecture of the Human Neocortex

**DOI:** 10.3389/fninf.2022.753770

**Published:** 2022-02-24

**Authors:** Ethan H. Kim, Derek Howard, Yuxiao Chen, Shreejoy J. Tripathy, Leon French

**Affiliations:** ^1^Krembil Centre for Neuroinformatics, Centre for Addiction and Mental Health (CAMH), Toronto, ON, Canada; ^2^Institute of Medical Science, Temerty Faculty of Medicine, University of Toronto, Toronto, ON, Canada; ^3^Department of Psychiatry, University of Toronto, Toronto, ON, Canada; ^4^Campbell Family Mental Health Research Institute, Centre for Addiction and Mental Health (CAMH), Toronto, ON, Canada

**Keywords:** neocortex, neuroinformatics, human brain (cerebral cortex), application (app), transcriptomics

## Abstract

The application of RNA sequencing has enabled the characterization of genome-wide gene expression in the human brain, including distinct layers of the neocortex. Neuroanatomically, the molecular patterns that underlie the laminar organization of the neocortex can help link structure to circuitry and function. To advance our understanding of cortical architecture, we created *LaminaRGeneVis*, a web application that displays across-layer cortical gene expression from multiple datasets. These datasets were collected using bulk, single-nucleus, and spatial RNA sequencing methodologies and were normalized to facilitate comparisons between datasets. The online resource performs single- and multi-gene analyses to provide figures and statistics for user-friendly assessment of laminar gene expression patterns in the adult human neocortex. The web application is available at https://ethanhkim.shinyapps.io/laminargenevis/.

## Introduction

RNA sequencing has provided molecular markers of human brain anatomy by revealing spatial gene expression patterns. The application of these techniques has provided genome-wide profiles of expression, but the brain’s complexity has limited our understanding of its cellular, molecular, and laminar architecture. Specifically, while the cytoarchitecture of the recently evolved neocortex has been characterized, we lack a strong understanding of its layer-specific gene expression.

Currently, few web applications can be used to visualize gene expression in the adult human neocortex. The Allen Brain Atlases from the Allen Institute of Brain Science (AIBS) and other tools allow viewing spatial expression patterns (Hawrylycz et al., 2012; Shen et al., 2012; Zeng et al., 2012; Guo et al., 2019; Maynard et al., 2021). These web applications can be used to examine laminar expression profiles and the regional variation that is associated with laminar differences across the cortex. However, there are no visualization tools to analyze expression across human neocortical layers for multiple datasets. Several datasets provide this laminar data but due to differences in the scopes and methods used, accessing and comparing this data is difficult and time-consuming. Here, we present *LaminaRGeneVis*, a web application for analyses of gene expression across human neocortical layers. *LaminaRGeneVis* enables visualization and analysis of data from layer-specific bulk-tissue, single-nucleus, and spatial transcriptomic RNA sequencing studies.

## Data and Methods

### Datasets

We used data from three studies that assayed genome-wide expression across the layers of the human neocortex in neurotypical donors. First, He and colleagues transversely sliced dorsolateral prefrontal cortex samples (DLPFC) from postmortem brains (He et al., 2017). Guided by their analyses, we focused on their first dataset (DS1) which contains expression data from four brains and was obtained from SRA using project code SRP065273. A second study of the DLPFC from three adult donors employing the spatial transcriptomics 10× Genomics Visium platform was obtained from the spatialLIBD R package (Maynard et al., 2021). The third dataset is from AIBS and assayed expression with single-nucleus RNA sequencing (snRNA-seq) in three brains^1^. We chose to use data only from the middle temporal gyrus, as this region had the most samples. The data was also split into three cell types as labeled by AIBS: GABAergic, glutamatergic and non-neuronal. While methods for spatial dissection vary, all three of these studies employed RNA sequencing and profiled the adult human neocortex. The characteristics of these datasets are described in Table 1. Similarity analyses were performed on the datasets to validate subsequent expression correlation and layer-specific enrichment analyses. The results of those analyses are available in the Supplementary Material.

**TABLE 1:** Characteristics of the datasets used in analysis.

Dataset	Technique used	Type of samples used:	Gene expression quantification	Cortical region assayed:	# of genes assayed	# of donors
He et al.	Illumina RNA-seq	Bulk-tissue	Gene count	PFC (BA 9, 10)	59,453	4 M
Maynard et al.	10× Visium	Tissue sections	Raw UMI count	DLPFC (BA 46)	18,633	2 (1 M, 1 F)
Allen cell type database	SMART-seq snRNA-seq	Single nuclei	Gene count	MTG	50,286	3 (2 M, 1 F)

*PFC, prefrontal cortex; DLPFC, dorsolateral prefrontal cortex; MTG, middle temporal gyrus; BA, Brodmann area; UMI, unique molecular identifier; M, male; F, female.*

#### Dataset Processing

**FIGURE 1:**
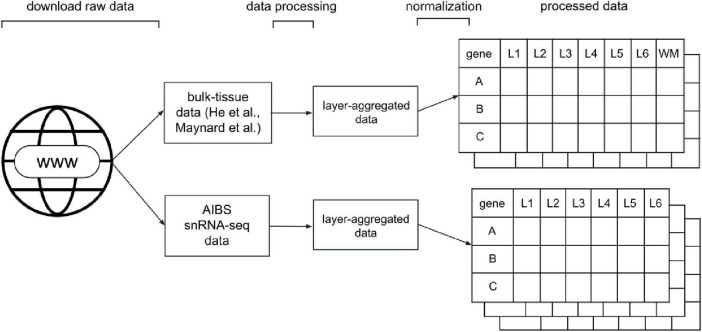
Diagram of the data processing pipeline. For the He and Maynard data, the standardization and processing stages result in an expression matrix of genes (rows) by layers, from layer 1 (L1) to 6 (L6) or white matter (WM). For the snRNA-seq data, the pipeline results in three gene expression matrices for each major cell-type label provided by the AIBS (GABA: GABAergic, GLUT: glutamatergic, NONN: non-neuronal).

To be able to compare the varying types of data, we processed and standardized the data such that each dataset was represented in a gene expression matrix. Our processing resulted in five such matrices, one each from the He et al. and Maynard et al. studies, and three for the AIBS snRNA-seq data, one for each major cell-type label they provided (GABAergic, glutamatergic and non-neuronal cells). Gene expression was represented in a gene-by-layer expression matrix as counts per million (CPM). A generalized visual schematic of our data processing pipeline is shown in Figure 1. We detail the processing of each dataset, as well as a summary of the source data, below.

#### Bulk-Tissue Data

The first of the bulk-tissue datasets is the He et al. study, where the authors collected samples from the PFC from each of the six male donors. From those larger tissue samples, they dissected out 18 50 micron thick slices parallel to the layers of the brain. Each section was then mapped back as belonging to a specific cortical layer and homogenized for RNA sequencing on the Illumina HiSeq platform. There were two resulting datasets: Dataset 1 and 2. Guided by the analyses from the source study, we utilized data from Dataset 1, which contained four male donors of the total six available.

We downloaded the raw RNA-seq data from He et al. from the Sequence Read Archive, project code SRP065273 in.fastq format. Genome alignment was performed using STAR 2.7 (Dobin et al., 2013) against the reference genome GRCh38.p13 with the corresponding annotation file from Ensembl (Yates et al., 2020). Default parameters were used. Aligned reads were quantified using RSEM (Li and Dewey, 2011) against the same reference genome and annotation file. This created a count matrix of 59,453 genes across 102 samples. In instances where multiple Ensembl ID’s referred to the same HGNC gene symbol, we took the average of the counts. For each of the samples that were labeled as the same transverse slice (e.g., all samples with the S1 label for slice 1), we summed the counts across the sample columns for all 18 transverse slices. The resulting 18 columns were pseudo-bulked at the layer level to create seven columns representative of the six layers of the human neocortex and one white matter layer using the mapping provided by He and colleagues (weighted averages). To avoid taking the log of 0, we added 1 to all counts prior to counts per million (CPM) normalization. We used the cpm() function from the edgeR package (Robinson et al., 2010; McCarthy et al., 2012) to CPM normalize the data, using log = T to log_2_ transform the data. The final normalized expression matrix contains 59,453 genes’ expression in the six neocortical layers and one layer of white matter in the PFC.

#### Spatial Transcriptomics Data

The second study, which employed a spatial transcriptomic assay is the Maynard study, which took coronal samples from the DLPFC from two male donors and one female donor (Maynard et al., 2021). From each coronal sample, they took four samples, termed “spatial replicate samples” by the authors, that spanned the cortical layers. Each of the replicate samples was run through the 10X Genomics Visium platform, where tens of thousands of small samples, termed “spots,” were assayed per sample using specialized slides proprietary to the platform. Each of the spots has the capability of assaying the whole genome. The result of their data processing pipeline, as well as the raw data from the Visium platform, is available for use as a package in the R programming language.

To access the data from their study, we used the fetch_data() function in their package with type = “sce_layer” to download the sce_layer data, which is the overall matrix containing gene expression data for 22,331 genes across the three donors and their 12 replicate samples spanning the six neocortical layers and one white matter layer. We only used the samples from two of the three donors (sample ID’s: 151507, 151508, 151509, 151510, 151673, 151674, 151675, and 151676) that contained data for all six cortical layers, reducing the number of usable replicate samples to 8. In contrast to the He et al. data, gene expression was represented in raw unique molecular identifier (UMI) counts. After examining the count data, we determined that the methods used to aggregate and normalize the He et al. data would be appropriate. The final matrix contains 18,633 genes’ expression in the six neocortical layers in the DLPFC, as well as a layer of white matter similar to the He et al. matrix.

#### snRNA-seq Data From the Allen Institute of Brain Science

The single nuclei data from the AIBS was generated by taking samples from multiple cortical regions, such as the anterior cingulate cortex and the middle temporal gyrus among others, across three donors. Samples of target areas were taken from coronal sections of the brain and each layer was dissected out. These layers were homogenized and labeled with DAPI and NeuN to identify if the cells were neuronal, or non-neuronal, respectively. The homogenized sample was then run through flow cytometry to separate out DAPI and NeuN-positive nuclei. Once separated, the nuclei were sequenced by Illumina HiSeq to quantify gene expression. The cells were given a layer label and cell type labels. From the areas available, we chose to use only the middle temporal gyrus due to the largest abundance of nuclei (*n* = 15,519) sampled in the dataset. This data is available on their Allen Brain Cell Types database under the “Multiple Cortical Area – SMART-seq (2019) dataset.”

We accessed the AIBS data from https://portal.brain-map.org/atlases-and-data/rnaseq/human-multiple-cortical-areas-smart-seq. The count matrix provided nucleus-level data, with cell-type and layer labels per nucleus. Using the provided metadata, we first filtered out samples with outlier_call = TRUE to remove any outlier nuclei. We then selected nuclei sampled from the middle temporal gyrus (MTG). Of those nuclei, we randomly downsampled the data such that per major cell type label provided (GABAergic, glutamatergic, non-neuronal), it contained the same number of nuclei per layer. This step was taken to equalize the sparsity of lowly expressed genes. After downsampling, the count matrix was pseudo-bulked at the level of layers by summing the raw gene-expression counts across all samples for each layer, cell type and gene. The bulked dataset was normalized through CPM normalization and log_2_-transformation, similar to the He and Maynard datasets. We then separated the dataset by cell type labels (GABAergic, glutamatergic, non-neuronal) to create three distinct normalized expression matrices of 50,286 genes across the 6 neocortical layers of the MTG.

### Statistical Analyses

#### Gene Expression Correlation

Pearson correlation was used to assess agreement for single genes between the Maynard and He datasets. This is calculated using a given gene’s seven layer-specific expression values from the He and Maynard datasets. For multiple genes, average correlation is used. To add a genome-wide perspective, the percentile of these correlations in reference to all other gene-to-gene values are also calculated.

#### Layer-Specific Gene Set Enrichment

To test for the enrichment of a set of genes in a given layer, we first removed genes with CPM < 0.1 across all layers, and normalized through log2-transformation then z-score normalized. We sorted the normalized expression matrices per layer by ranking the remaining genes in each dataset from the most to least normalized expression. Within this ranking, the area under the receiver operating characteristic curve (AUC) was used to test whether the inputted set of genes were enriched or depleted (enrichment: AUC > 0.5, depletion: AUC < 0.5). The Mann–Whitney *U* test was used to determine statistical significance and multiple-test corrected with Bonferroni correction.

#### Availability of Data and Code

Data used in the application and the code to process the data are available online at https://github.com/ethanhkim/laminargenevis. Scripts to process the raw data from He et al. are available at https://github.com/derekhoward/he_seq.

## Results

**FIGURE 2:**
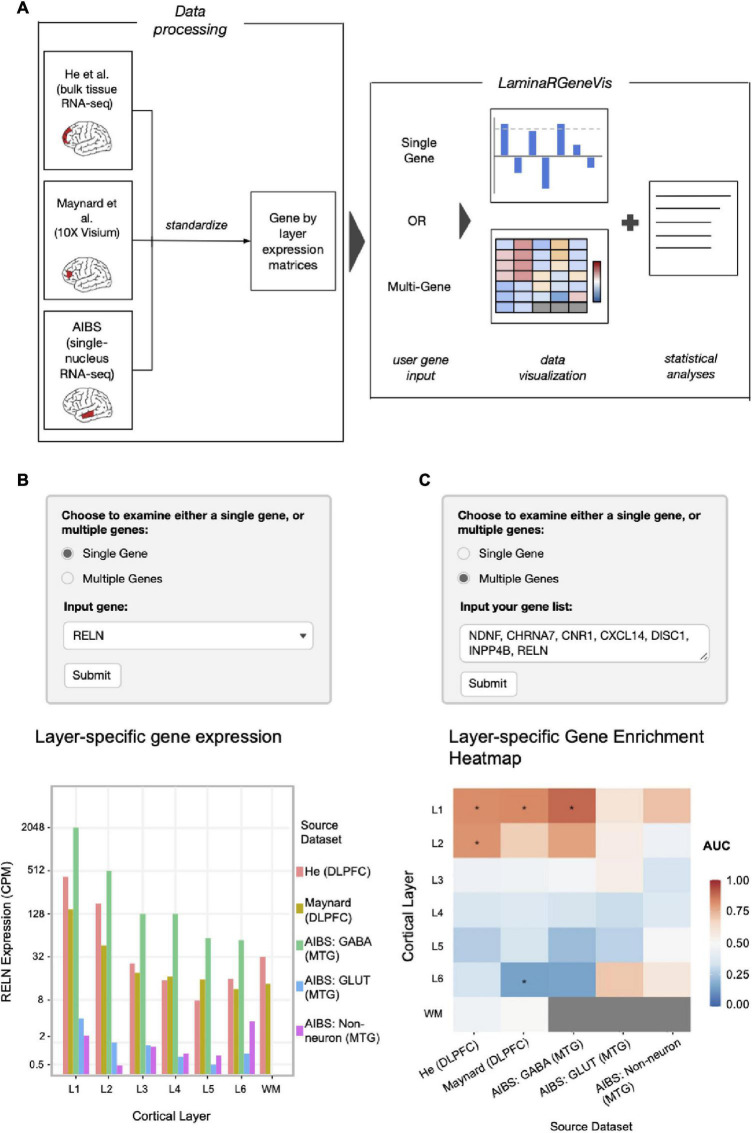
**(A)** Overview diagram of the data processing steps and web application interface combined. **(B)** Example single-gene input settings and output for *RELN* expression (CPM on a log scaled *y*-axis) across the cortical layers and white matter with color marking the source datasets and cell types. **(C)** Example multi-gene input settings and heatmap visualization output for genes found to mark layer 1 in a separate study of laminar expression patterns (Zeng et al., 2012). Cells are colored according to the enrichment of layer-specific expression of the input genes (AUC scores). *P*-values were calculated using the Mann-Whitney *U* test and adjusted for multiple test correction through Bonferroni correction; asterisks (*) indicate p_*corrected*_ < 0.05.

We have developed an online web application, *LaminaRGeneVis*, that can be accessed at https://ethanhkim.shinyapps.io/laminargenevis/. The application allows users to visualize layer-specific gene expression in the human neocortex using the aforementioned processed data from He et al., Maynard et al. and the AIBS. Furthermore, the application will also report statistical analyses that assay agreement and test layer-specific enrichment for a set of genes. As shown in Figure 2A, there are two main “modes” of analyses: “Single Gene,” where a user can examine the expression of a single gene across our curated datasets, and “Multiple Genes,” where a user can review the expression of multiple genes.

### Single Gene Mode

The user can input their gene of interest by typing in its gene symbol and selecting it from the drop-down list. Once submitted, the gene’s normalized expression across the cortical layers in each dataset is displayed as a bar plot. A text box below notes which datasets assayed the queried gene and the agreement statistics between the He and Maynard datasets. Agreement statistics that compare the snRNA-seq expression profiles are not provided because our genome-wide comparisons found much weaker cross-dataset correlations when compared to the comparisons between the He and Maynard datasets (Supplementary Results, Tables 2, 3).

An example barplot of a single gene’s expression profile is given in Figure 2B. As reported by the tool, the expression correlation between the He and Maynard datasets for *RELN* is 0.916 (*p* = 0.0038). To provide a genome-wide perspective, a ranking is also provided (91st quantile for *RELN*). We chose *RELN* to demonstrate the tool due to its known layer specificity (Martínez-Cerdeño and Clascá, 2002). In agreement with the plotted values, it was found to mark layer I in the He et al. and Maynard et al. datasets (also reported by *LaminaRGeneVis*). Thus, both the barplot and correlation value provide users with an understanding of a given gene’s expression pattern and consistency across the human neocortical layers.

### Multi-Gene Mode

In the Multi-gene mode, the application generates visualizations for the queried gene set’s layer-specific enrichment and normalized expression of each gene across datasets. Layer-specific enrichment is first visualized as a heatmap that displays the AUC values across layers and datasets. Normalized gene expression is additionally displayed as a heatmap for 30 genes or less and a dot plot otherwise. The dot plots also show the queried genes’ median expression in each layer. Finally, a summary textbox at the bottom reports information such as the number of inputted genes assayed in each dataset and agreement statistics.

An example output of multiple genes’ expression profiles is given in Figure 2C. This heatmap is the first visualization shown and summarizes enrichment across datasets. The genes used as input were found to mark layer 1 in a separate study of laminar expression patterns (Zeng et al., 2012). This study examined *in situ* gene expression patterns of ∼1,000 genes in the human temporal and visual cortices. Reassuringly, these seven marker genes highlight layer 1 in the He and Maynard datasets that profiled the prefrontal cortex (both p_*adjusted*_ < 0.03). The following five figures provided by the tool expand upon this summary heatmap by providing per dataset heatmaps that mark the expression of individual genes. These plots mark *DISC1* as having discordant expression between the He and Maynard datasets. In agreement, heterogeneous expression of *DISC1* was also noted by Zeng and colleagues (Zeng et al., 2012). Like the Single Gene mode, the tool correlates laminar expression profiles across the datasets. For these seven marker genes the mean correlation is 0.866 (*p* = 0.0026, *n* = 6 layers). Genome-wide, this correlation ranks in the 81st quantile in all single correlation values, suggesting a high degree of bulk-tissue dataset agreement. The given correlation values and the visualizations provide multiple perspectives of a given gene set’s expression across the laminar architecture of the human neocortex.

**FIGURE 3:**
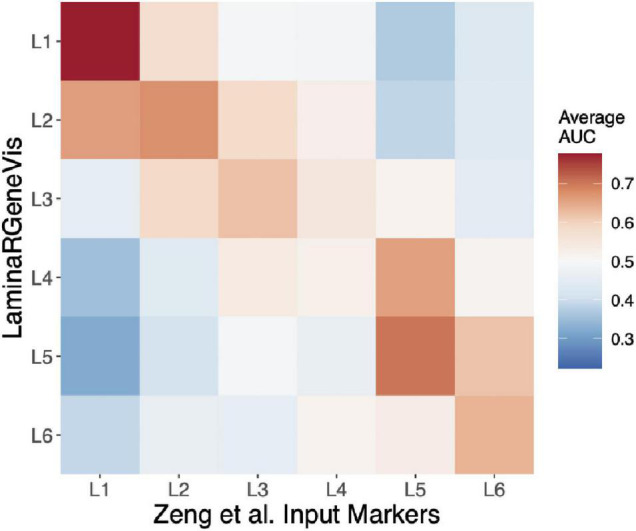
Heatmap visualization of the *LaminaRGeneVis* enrichment AUC values for the laminar specific expression markers provided by Zeng and colleagues (*x*-axis). Each column displays the average AUC enrichment values across the five datasets from layer 1 (L1) to 6 (L6).

To further test our tool beyond the layer 1 markers, we used each set of the Zeng et al. laminar markers as input. These markers were identified from an independent experimental method (*in situ* hybridization), and their survey examined two regions, one of which was not profiled in the *LaminaRGeneVis* datasets (visual cortex). As shown in Figure 3, we summarize the six sets of results with a single heatmap that averages the AUC values across the five datasets for a given input set of marker genes (Figure 3). Although the absolute average mean AUC value varies across the marker sets, the correct layer had the highest AUC value for all layers except the fourth. In summary, this agreement across the layers and datasets further validates the ability of *LaminaRGeneVis* to assess laminar expression patterns.

## Discussion

With the advances in sequencing and imaging techniques, the ability to assay and image the human brain has become increasingly more cost-efficient and powerful. This has led to more studies examining the genome-wide expression profiles of the brain. The resulting datasets have allowed researchers to analyze aspects of neuroanatomy which were not possible before these developments, such as cell-type-specific differences in gene expression.

In lieu of a centralized platform to compare neuroanatomical gene expression datasets, *LaminaRGeneVis* is beneficial to researchers in a few ways. First, genes related to neurological or neuropsychiatric disorders can be examined in a layer-specific manner. Such layer-specific analyses are motivated by neuropathological findings: thinning of layers III and V of the frontal lobe have been observed in patients with schizophrenia (Rajkowska et al., 2002; Williams et al., 2013; Wagstyl et al., 2016) and the neuronal density in multiple cortical layers in various cortical regions have been reported as being lower in patients with major depressive disorder (Rajkowska et al., 1999), which is consistent with suicide cases (Underwood et al., 2012); however, it is unclear why those specific layers are more vulnerable. Characteristics of these layers, such as their cell-type proportions and transcriptomic fingerprints, can assist in understanding the neural underpinnings of these disorders. Researchers studying major depressive disorder, for example, could input their set of disorder-related genes into our tool to easily visualize and quantify expression across the cortical layers.

That leads to another benefit of our tool, which is comparisons across datasets. As outlined, there are differences in accessibility that make comparisons between datasets difficult and time-consuming. Additionally, by using three datasets, we increase the sample size to cover nine human brains. While all these datasets provide genome-wide expression patterns across the layers, they use different expression profiling techniques and cortical regions, which allows users to extract robust patterns. While *LaminaRGeneVis* is not a tool to conduct an extensive meta-analysis, it provides easy-to-understand and straightforward visualizations and analyses that join datasets.

However, our application is not without limitations. Its most prominent limitations come from the data used in the application. The data are sourced from different regions in the brain: He et al. and Maynard et al. profiled the frontal lobe, while the snRNA-seq data from AIBS used here is sampled from the temporal lobe. It is well known that the thickness of the cortical layers varies throughout the cortex (Brodmann, 1909; von Economo et al., 2008), as well as cell-type-specific differences (González-Acosta et al., 2018). This limited set of cortical regions with laminar-specific expression limits the generalization of the tool’s results. For example, there may be genes with laminar-specific expression in the parietal cortex that are homogeneously expressed across the layers of the prefrontal and temporal cortices due to regional differences in cytoarchitecture. However, our tests of the Zeng et al. markers that were partially obtained from the visual cortex provide some support for regional consistency. Another limitation comes from the sample processing methods used across the datasets. For example, by isolating nuclei, the AIBS data does not capture expression in the cell soma, axons and dendrites. Recent evidence suggests that there are relatively distinct transcriptomes in neuronal dendrites (Perez et al., 2021) and axons (Maciel et al., 2018). By visualizing snRNA-seq, spatial transcriptomic, and bulk-tissue RNA-seq data, *LaminaRGeneVis* combines datasets. Finally, there is a neuronal bias in the snRNA-seq AIBS data because they profiled fewer non-neuronal than neuronal nuclei. Glial cells make up perhaps an equal, if not greater, proportion of the total cells in the brain compared to neuronal cells (von Bartheld et al., 2016). Due to these differences, the tool clearly displays the cortical regions and if data is from single nuclei or tissue sections. This allows the user to gauge region- and technique-specific effects. We also limit statistical comparisons to the He and Maynard datasets that assay expression in the same region. We look forward to adding data from additional transcriptomic studies to address these limitations.

## Conclusion

We have developed a web application for visualizing gene expression across the laminar architecture of the adult human neocortex. It reports cross-dataset correlation and the enrichment of layer-specific expression. These functionalities provide easily accessible figures and statistics for quick assessment of expression across the layers of the human cortex.

## Data Availability Statement

Data used in the application and the code to process the data are available online at https://github.com/ethanhkim/laminargenevis. Scripts to process the raw data from He et al. are available at https://github.com/derekhoward/he_seq.

## Author Contributions

EK and LF contributed to the conception of the study. ST helped refine the design of the study. EK performed the dataset processing, normalization and the creation of *LaminaRGeneVis*, and wrote the first draft of the manuscript. DH and YC implemented the processing pipeline for the He et al. dataset. EK, LF, and ST edited the manuscript. LF and ST supervised the research. All authors contributed to the drafting of the manuscript.

## Conflict of Interest

The authors declare that the research was conducted in the absence of any commercial or financial relationships that could be construed as a potential conflict of interest.

## Publisher’s Note

All claims expressed in this article are solely those of the authors and do not necessarily represent those of their affiliated organizations, or those of the publisher, the editors and the reviewers. Any product that may be evaluated in this article, or claim that may be made by its manufacturer, is not guaranteed or endorsed by the publisher.

## Footnotes

1
https://portal.brain-map.org/atlases-and-data/rnaseq/human-multiple-cortical-areas-smart-seq
