# Supplementary material for: LaminaRGeneVis: A Tool to Visualize Gene Expression Across the Laminar Architecture of the Human Neocortex
Source: Front Neuroinform. 2022 Feb 24;16:753770. doi: 10.3389/fninf.2022.753770 (PMC8907970; doi:10.3389/fninf.2022.753770)
Supplement: Supplementary file 1 [file Data_Sheet_1.docx]

# ***Supplementary Material***

## **1 Supplementary Methods**

We evaluated dataset similarity using the Mantel test (Mantel, 1967) through the mantel() function in the vegan R package (Oksanen et al., 2020). To compare any two datasets, they were subset by a list of genes common to both datasets. As Mantel tests compare the similarity between two correlation matrices, each dataset is correlated to itself using the corr() function with method = “pearson” as a parameter to create gene expression correlation matrices required for Mantel testing (Supp. Fig. 1A). The mantel() function transforms each of the correlation matrices into one singular vector of gene-to-gene correlation values (Supp. Fig. 1B). The resulting vectors are correlated through a Pearson correlation test using “pearson” as a parameter within the mantel() function (Supp. Fig. 1C). This results in a Mantel r statistic that ranges from -1 to 1, with -1 indicating a total negative correlation, 0 indicating no relationship and 1 indicating total positive correlation.

## **2 Supplementary Results**

We evaluated dataset similarity using the Mantel test (Mantel, 1967). We used the implementation provided by the vegan R package (Oksanen et al., 2020), using Pearson correlation as the statistical method. As the Mantel test correlates similarity or distance matrices, We used gene-gene Pearson correlation matrices for the similarity matrices to test if across layer gene associations are consistent within and across the datasets.

We first examined the inter-and intra-donor similarity in the Maynard data for the two donors with all 6 cortical layers and white matter layer labelled. We downloaded the layer-level data as previously described, from which we separated each sample from the two donors and normalized through counts-per-million (CPM) normalization and log2-transformation. This resulted in 4 expression matrices per donor for a total of 8 matrices. The mean Mantel r statistic for donor 1 was 0.4106 and 0.4140 for donor 2. When comparing across donors, the mean r statistic was 0.2947. We validated these results by subsetting the Maynard data to a list of genes used by Zeng et al (Zeng et al., 2012). This resulted in a mean r statistic of 0.5303 for donor 1 and 0.6456 for donor 2. The mean r statistic across donors was 0.4324. All sample-to-sample comparisons can be found in Supplementary Table 1.

We then compared the similarity between the Maynard and He datasets using the bulked and normalized datasets as described in the main manuscript. We subsetted the datasets to a list of genes common between the two (n = 18,206), and each dataset was correlated to itself to create correlation matrices for Mantel testing. We found that the Mantel r statistic was 0.5166 when comparing across datasets. Removing genes with CPM < 0.1 prior to log-transformation resulted in the r statistic increasing to 0.5731 (15,589 genes). We improved these results by subsetting the two datasets to a list of layer-specific marker genes (Zeng et al., 2012). We found that the r statistic was 0.683 (946 genes) and 0.7246 when filtering for genes with CPM > 0.1 (882 genes).

We also compared the bulk-tissue datasets to the cell-type specific snRNA-seq data from the AIBS using the aforementioned processes for comparing the Maynard and He data. We found after removing white matter data from the bulk-tissue, there was little to no correlation in either direction between the bulk-tissue and cell-type specific snRNA-seq data: Comparing the He dataset against each of the cell-type specific data resulted in a mean r statistic of -0.08134 (30,744 genes) and 0.0007667 for Maynard et al. (17,197 genes). When filtering for genes with CPM > 0.1 across all layers, there were slight increases in the r statistic and subsetting to the list of genes from Zeng et al. also slightly increased the r statistic.


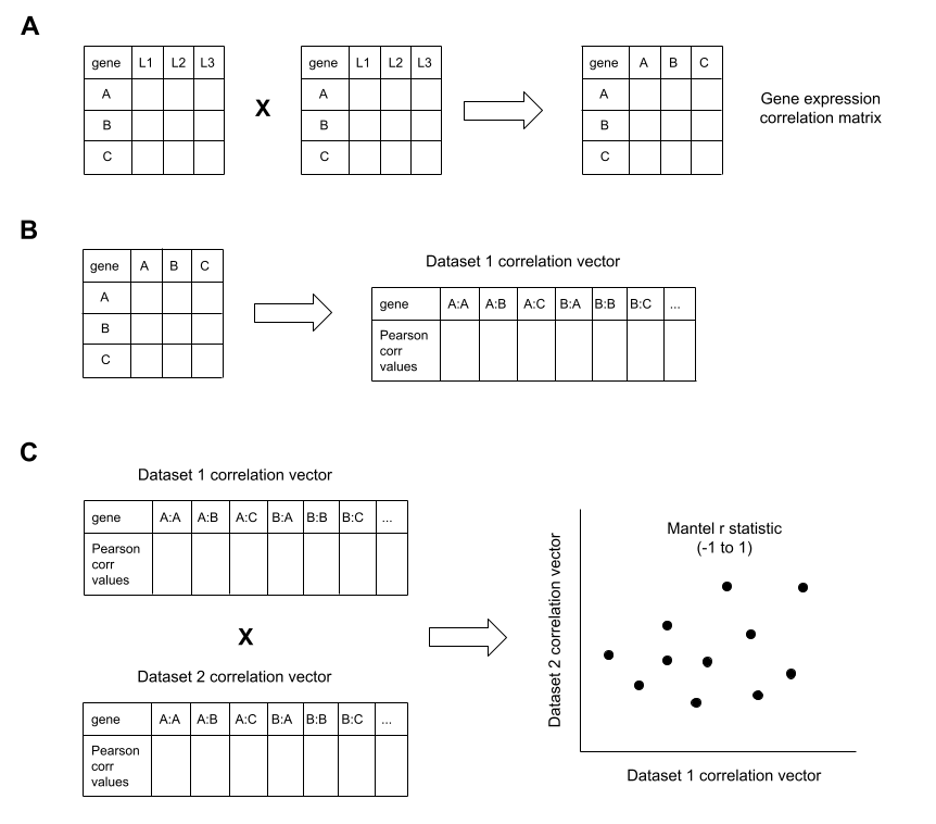


**Supplementary Figure 1**. Visual diagram for the Mantel test procedure performed by the mantel() function for the dataset similarity analysis. **(A)** A matrix of gene expression is correlated to itself via Pearson correlation testing to create a gene expression correlation matrix. **(B)** The correlation matrix is flattened into a correlation vector containing all gene-to-gene Pearson correlation values. **(C)** The steps from A and B are performed for each respective dataset, and the resulting correlation vectors are correlated against each other through Pearson correlation testing to give a Mantel r statistic which ranges from -1 (total negative correlation) to 1 (total positive correlation).

## **Supplementary Table 1: Dataset similarity testing results for Maynard et al data**

| **Donor label 1** | **Donor label 2** | **Sample IDs tested** | **Mantel statistic r** | **Mantel statistic r; filtered for Zeng et al marker list** |
| --- | --- | --- | --- | --- |
| 1 | 1 | 151507, 151508 | 0.3867437 | 0.4929938 |
| 1 | 1 | 151507, 151509 | 0.3761705 | 0.4928974 |
| 1 | 1 | 151507, 151510 | 0.3662345 | 0.5029497 |
| 1 | 1 | 151508, 151509 | 0.4472683 | 0.5596710 |
| 1 | 1 | 151508, 151510 | 0.4217159 | 0.5252885 |
| 1 | 1 | 151509, 151510 | 0.4652534 | 0.6079797 |
| 2 | 2 | 151673, 151674 | 0.4450776 | 0.6862513 |
| 2 | 2 | 151673, 151675 | 0.4396620 | 0.6846133 |
| 2 | 2 | 151673, 151676 | 0.4170934 | 0.6318101 |
| 2 | 2 | 151674, 151675 | 0.4021281 | 0.6449719 |
| 2 | 2 | 151674, 151676 | 0.3875977 | 0.6121642 |
| 2 | 2 | 151675, 151676 | 0.3922045 | 0.6138546 |
| 1 | 2 | 151507, 151674 | 0.3079286 | 0.4083163 |
| 1 | 2 | 151507, 151673 | 0.2747824 | 0.4145270 |
| 1 | 2 | 151507, 151675 | 0.2853121 | 0.4195137 |
| 1 | 2 | 151507, 151676 | 0.2729248 | 0.4080120 |
| 1 | 2 | 151508, 151674 | 0.3110389 | 0.3904035 |
| 1 | 2 | 151508, 151673 | 0.2602919 | 0.3850534 |
| 1 | 2 | 151508, 151675 | 0.2833523 | 0.3833158 |
| 1 | 2 | 151508, 151676 | 0.2703848 | 0.3671039 |
| 1 | 2 | 151509, 151674 | 0.3625330 | 0.5043142 |
| 1 | 2 | 151509, 151673 | 0.3021198 | 0.4848182 |
| 1 | 2 | 151509, 151675 | 0.3207013 | 0.4837968 |
| 1 | 2 | 151509, 151676 | 0.3041808 | 0.4514819 |
| 1 | 2 | 151510, 151674 | 0.3313964 | 0.4796648 |
| 1 | 2 | 151510, 151673 | 0.2823863 | 0.4555763 |
| 1 | 2 | 151510, 151675 | 0.3066032 | 0.4513501 |
| 1 | 2 | 151510, 151676 | 0.2389498 | 0.4309212 |

## **Supplementary Table 2: Dataset similarity testing results for Maynard et al, He et al and Allen Institute data**

| **Dataset 1** | **Dataset 2** | **Mantel statistic r** | **Mantel statistic r; filtered for CPM > 0.1** | **Mantel statistic r; filtered for Zeng et al list** |
| --- | --- | --- | --- | --- |
| He et al | ACTD - GABAergic | -0.1077 | -0.006967 | 0.03838 |
| He et al | ACTD - Glutamatergic | -0.1295 | -0.005718 | 0.03376 |
| He et al | ACTD - Non-neuronal | -0.006967 | 0.05965 | 0.07922 |
| He et al | ACTD - layer-aggregate | 0.03536 | 0.09435 | 0.1756 |
| Maynard et al | ACTD - GABAergic | -0.01428 | 0.002822 | 0.03121 |
| Maynard et al | ACTD - Glutamatergic | -0.0143 | 0.009769 | 0.064 |
| Maynard et al | ACTD - Non-neuronal | 0.02628 | 0.04014 | 0.06274 |
| Maynard et al | ACTD - layer-aggregate | 0.08748 | 0.1037 | 0.201 |
| He et al | Maynard et al | 0.5166 | 0.5731 | 0.683 |

** ACTD Allen Cell Type Database; MTG, middle temporal gyrus; BA, Brodmann Area*

##

##

## **References**

Mantel, N. (1967). The detection of disease clustering and a generalized regression approach. *Cancer Research*, *27*(2), 209–220.

Oksanen, J., Blanchet, G. F., Friendly, M., Kindt, P., McGlinn, D., Minchin, P. R., O’Hara, R. B., Simpson, G. L., Solymos, P., Steven, M. Henry. H., Szoecs, E., & Wagner, H. (2020). *vegan: Community Ecology Package* (2.5-7) [R]. [https://CRAN.R-project.org/package=vegan](https://cran.r-project.org/package=vegan)

Zeng, H., Shen, E. H., Hohmann, J. G., Oh, S. W., Bernard, A., Royall, J. J., Glattfelder, K. J., Sunkin, S. M., Morris, J. A., Guillozet-Bongaarts, A. L., Smith, K. A., Ebbert, A. J., Swanson, B., Kuan, L., Page, D. T., Overly, C. C., Lein, E. S., Hawrylycz, M. J., Hof, P. R., … Jones, A. R. (2012). Large-scale cellular-resolution gene profiling in human neocortex reveals species-specific molecular signatures. *Cell*, *149*(2), 483–496. https://doi.org/10.1016/j.cell.2012.02.052
